# Synthesis and Preparation of Chitosan/Clay Microspheres: Effect of Process Parameters and Clay Type

**DOI:** 10.3390/ma11122523

**Published:** 2018-12-12

**Authors:** Bárbara Fernanda F. dos Santos, Matheus Aleixo Maciel, Albaniza A. Tavares, Clarissa Q. B. de Araújo Fernandes, Wladymyr Jefferson B. de Sousa, Marcus Vinícius Lia Fook, Itamara Farias Leite, Suédina Maria de Lima Silva

**Affiliations:** 1 Postgraduate Program in Materials Science and Engineering, Federal University of Campina Grande, Campina Grande 58429-900, Brazil; nandasantos_babi@hotmail.com (B.F.F.d.S.); albaniza.alves@gmail.com (A.A.T.); clarissa.queiroz@hotmail.com (C.Q.B.d.A.F.); wladymyrjb@yahoo.com.br (W.J.B.d.S.); 2 Department of Materials Engineering, Federal University of Campina Grande, Campina Grande 58429-900, Brazil; matheus_alexo@hotmail.com (M.A.M.); marcus.liafook@certbio.ufcg.edu.br (M.V.L.F.); 3 Department of Materials Engineering, Federal University of Paraiba, João Pessoa 58051-900, Brazil; itamaraf@gmail.com

**Keywords:** chitosan, clay, microspheres, physical properties

## Abstract

This work aimed to prepare chitosan/clay microspheres, by the precipitation method, for use in drug carrier systems. The influence of the process parameters, particularly two airflows of the drag system (2.5 and 10 L·min^−1^) on the microspheres physical dimensions and properties, such as microstructure, degree of swelling and porosity were evaluated. The samples were characterized by optical microscopy (OM), scanning electron microscopy (SEM) and X-ray diffraction (XRD). Water absorption and porosity tests were also performed. The results showed that the process parameters affected the size of the microspheres. The diameter, volume and surface area of the chitosan/clay microspheres decreased when they were prepared with the higher airflow of the drag system. The microspheres presented a porous microstructure, being the pore size, percentage of porosity and degree of swelling affected not only by the process parameters but also by the type of clay. Hybrids (chitosan/clay) with intercalated morphology were obtained and the hybrid prepared with montmorillonite clay at higher airflows of the drag system presented the greatest interlayer spacing and a more disordered morphology. Thus, it is certain that the chitosan/clay nanocomposite microspheres prepared with montmorillonite (CL clay) at higher airflows of the drag system can have good drug-controlled release properties.

## 1. Introduction

Chitosan is a biopolymer, derived by the deacetylation of chitin, which consists of D-glucosamine and N-acetyl-D-glucosamine units and is frequently used as excipient in pharmaceutical dosage forms [1,2,3,4]. Chitosan has been widely used since it exhibits a number of unique properties such as biocompatibility, biodegradability and non-toxicity [5]. It is a weak base with pKa in the range of 6.2–7.0, being insoluble in water at neutral and alkaline pH. However, chitosan is soluble at acidic pH, since the amino groups are protonated. Importantly, positively charged chitosan can interact with polyanions and form crosslinked matrices [6,7,8,9]. Furthermore, positively charged chitosan can form polyelectrolyte complexes with polyanionic macromolecules, such as alginate [10], gelatin [11], carboxymethylcellulose [12] and also with anions as sulfate, citrate tripolyphosphate (TPP) ions and clays minerals [13,14] and can be widely used in the controlled release of drugs into the stomach orally.

It has to be pointed out that this type of polyelectrolyte complex formation can significantly alter key properties of the respective drug delivery systems, including for instance their morphology, drug encapsulation efficiency and drug release kinetics. Therefore, the addition of polyanionic compounds to chitosan based microspheres can be an interesting tool to adjust desired key features [15]. Clay minerals are natural cationic exchangers and thus can bind with cationic drugs in solution via electrostatic interaction. Depending on the cation exchange capacity of the clay, the cationicity of the drug and pH of the release medium determine the kinetics of drug release. Apart from electrostatic force, there also exist the possibility of other interactions, including hydrophobic, hydrogen bonding, ligand exchange and water bridging. These properties have encouraged the use of clay minerals for sustained release of drugs and improved drug dissolution [16,17,18,19,20]. Importantly, the different layers of clay can be separated upon hydration and clay can be negatively charged at the surface layers, due to the presence of -SiO- groups. This is why clay may interact with positively charged drugs [21,22], potentially resulting in sustained release from such complexes. Furthermore, the negative charges of silicate layers may also interact with positively charged macromolecules (e.g., chitosan), forming nanocomposite materials [23,24,25].

Chitosan/clay hybrids have great potential in controlled release formulations due to the various benefits that can be achieved with this association, among them: (a) the intercalation of cationic chitosan in the expandable aluminosilicate structure of clay is expected to neutralize the strong binding of cationic drug by anionic clay; (b) the solubility of chitosan at the low pH of gastric fluid will decrease and premature release of the drug in the gastric environment can be minimized; (c) cationic chitosan provides the possibility of efficiently loading negatively charged drugs compared with clay; and (d) the presence of reactive amine groups on chitosan provides ligand attachment sites for targeted delivery. The limited solubility of a chitosan–clay hybrids drug carrier at gastric pH offers significant advantages for colon-specific delivery because some drugs are destroyed in the stomach, at acidic pH and in the presence of digestive enzymes. Furthermore, the mucoadhesive property of chitosan can enhance the bioavailability of drugs in the gastrointestinal tract [26]. However, to achieve these benefits, issues as type of chitosan, type of clay, chitosan/clay ratio, crosslinking density between chitosan and clay, as well as, the type of delivery system (tablets, beads, films, nanoparticles, conjugates, hydrogels, capsules and microspheres) [27] along with their methods of preparations should be evaluated [28,29,30].

The practice of microsphere based delivery system allows control over drug release profile and specific target site by carefully tailoring the formulation of various polymer drug combinations [31]. This type of delivery system may be used for administration of drugs for localized action [32]. In addition, due to a high surface to volume ratio, this type of delivery system may present efficient absorption and enhanced bioavailability of the drugs [31]. Different methods for the formation of microspheres includes interaction with counter ions like anions (sulphate, phosphates, hydroxides), crosslinking, solvent evaporation, ionic gelation, spray drying, emulsion polymerization and precipitation/coacervation and so forth [31,33,34,35,36,37]. However, since these processes present high cost, Dias, et al. [21] and Prado, et al. [22] performed studies on construction of an inexpensive apparatus for the production of chitosan microspheres. Nevertheless, this apparatus has limitations in controlling the chitosan solution injection flow, because the system provides a pressure gradient between the beginning and the end of the process. In order to overcome these limitations, our research group developed an inexpensive apparatus for fabricating bioadhesive microspheres, based on chitosan for controlled release systems, using an automated chitosan solution injection flow system. According to results [38], the manufacturing parameters, injection and airflow rates, determine the microsphere particle-size distribution. By modulating these parameters, it was possible to produce chitosan microspheres with controlled size. The chitosan microspheres obtained were homogeneous in size with small deviation, especially when the smaller injection and airflow rates were used. However, the preparation of chitosan/clay microspheres using this inexpensive apparatus, has not been studied yet. Therefore, the objective of this work was to prepare chitosan/clay microspheres, by the precipitation method, using an inexpensive apparatus, aiming at the use in drug carrier systems. The influence of the process parameter (airflow in the drag system) and the clay type on the dimensions and physical properties of the microspheres, such as microstructure, degree of swelling and porosity, was evaluated.

## 2. Materials and Methods

### 2.1. Materials

Medium molecular weight chitosan from shrimp shells, Mv = 114 kDa determined by viscometry [39] and degree of deacetylation DD = 92 % determined by infrared spectroscopy method [40] was supplied by Polymar-Fortaleza/CE-Brazil (Fortaleza, Brazil). Sodium containing natural montmorillonite take-off with the trade name of Cloisite Na^+^ (CL) was provided by Southern Clay Products Inc. (Gonzales, TX, USA) and Argel bentonite clay (AG) was supplied by Bentonit União Nordeste-São Paulo/SP-Brazil (São Paulo, Brazil). The CL clay presents a cation exchange capacity (CEC) of 92.6 meq/100 g, given by the supplier and the AG clay of 92 meq/100 g, as determined by our research group in an earlier study [41]. These clays were used without any further purification. Glacial acetic acid and sodium hydroxide were provided by Vetec Química-Brazil (Duque de Caxias, Brazil) and sodium acetate was supplied by Nuclear-Brazil (Angra dos Reis, Brazil). All aqueous solutions were prepared using distilled water and all reagents and solvents were analytical grade and used as received.

### 2.2. Preparation of Chitosan/Clay Microspheres

**Figure 1:**
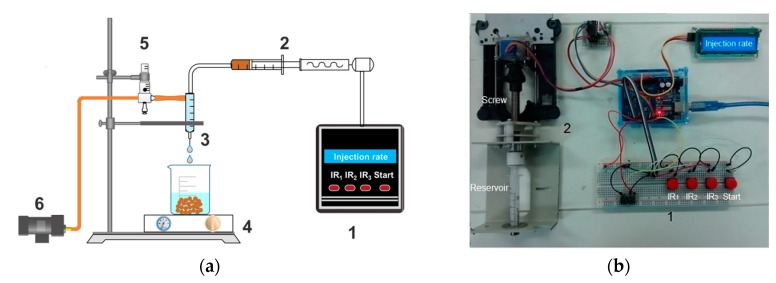
(**a**) Schematic diagram of the experimental equipment, used to prepare chitosan microspheres, developed in our lab: (1) electronic microcontroller, (2) injection zone, (3) dripper, (4) stirrer, (5) rotameter and (6) pump; (**b**) The magnification, items 1 and 2, of the experimental equipment, used to prepare chitosan microspheres.

Chitosan/clay microspheres were prepared by the precipitation method, employing experimental equipment developed in our laboratory [38] (Figure 1). Briefly, chitosan solution (4% *w*/*v*) was prepared using an aqueous solution of acetic acid (5% *v*/*v*) containing 4% of sodium acetate under magnetic stirring at room temperature (23 ± 2 °C) for 2 h. After this time, the pH of the solution was adjusted to 4.9, with the addition of a 1 M NaOH solution and then a clay suspension, previously prepared, was slowly added to chitosan solution in appropriate amount to reach a final clay concentration of 10 wt.% (based on chitosan weight). The chitosan/clay mixtures were maintained under mechanical stirring at 600 rpm and 50 ± 2 °C for 4 h and 30 min. Afterwards, the chitosan/cay mixtures were added dropwise, through a drip system constructed from polymeric pipe and a 0.45 mm diameter nozzle, into a gently stirred coagulation liquid (aqueous solution of NaOH, 8% *v*/*v*). The effects of manufacturing parameters on the characteristics of the resulting microspheres were studied by setting the injection flow rate at 0.150 mL⋅min^−1^ and the airflow rate of the drag system at 2.5 and 10.0 L⋅min^−1^. The formed microspheres suspension was filtered and washed with distilled water until neutrality and then dried in an oven at 50 °C for 24 h. Moreover, from time to time, the reactions of same sets of parameters were duplicated and the reproducibility was found to be excellent. Chitosan microspheres were manufactured by the same method and used for comparison purposes.

### 2.3. Characterization

#### 2.3.1. Determination of the Dimensions of Chitosan/Clay Microspheres

Optical microscopy (OM) of the chitosan and chitosan/clay microspheres was conducted in a microscope model Q734ZT series 059 from DP Scientific Instruments (Cambridge, MA, USA). The micrographs obtained were used to determine the diameter and volume of the prepared microspheres. To this end, a small amount of dried microspheres (about 10 microspheres) was placed on a clean glass slide. The slide containing the microspheres was allocated to the base of the microscope and the images obtained were analyzed using the software Pixcavator 6.0.

#### 2.3.2. Microspheres Morphology

The surface topography of the microspheres was examined under a scanning electron microscope (Shimadzu SSY-550, Tokyo, Japan). A small amount of dry microspheres, at least 10, was placed on aluminum stubs and made electrically conductive by coating with a thin layer of gold. A scanning electron photomicrograph was taken at the acceleration voltage of 30 kV, chamber pressure of 0.6 mm Hg. The images obtained were analyzed using the software Gwyddion 2.50.

X-ray diffraction (XRD) analysis of the chitosan and chitosan/clay microspheres, prepared with 10% by weight of clay, were conducted in Shimadzu XRD-7000 apparatus (Shimadzu, Tokyo, Japan), using copper Kα radiation (1.5418 Å), in a range of 2*θ* between 2 and 70 degrees, voltage of 40 kV, current 30 mA and speed of 1°/min. The basal spacing (d001) value of the pristine clay was determined by Bragg’s law, according to Equation (1) [42]. With this technique, it is possible to confirm the intercalation of the chitosan in the clay galleries by the expansion of basal spacing this one and to investigate if a microcomposite or nanocomposite was produced.

(1) $$d_{001}=\frac{8,8264273}{2\theta }$$

where *θ* is the measured diffraction angle in degree.

#### 2.3.3. Water Absorption

Water absorption was determined according to Depan, et al. [43]. Briefly, the chitosan and dry chitosan/clay microspheres of known mass were immersed in distilled water at 25 °C for 1 day (24 h). After this time, the microspheres were removed, quickly placed on absorbent paper, to remove surface water and then weighed. The water absorption percentage (A) of these samples was calculated using the Equation (2):

(2) $$A(\%)=\frac{\left(m-m_{0}\right)}{m_{0}}\times 100$$

where m and m_0_ are the masses of the wet and dry samples, respectively.

## 3. Results and Discussion

### Optical Microscopy

**Figure 2:**
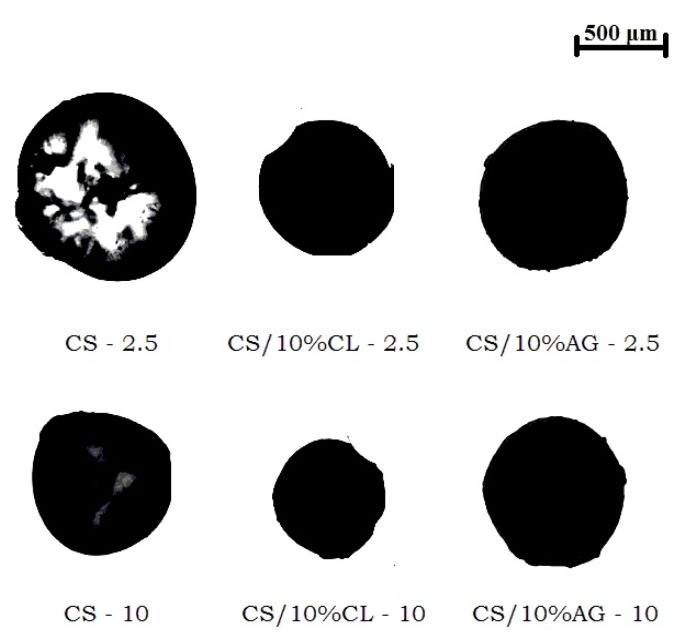
Optical microscopy mages of chitosan and chitosan/clay microspheres, with an injection flow of 0.150 mL·min^−1^ and airflows of 2.5 and 10 (L·min^−1^).

The images of chitosan and chitosan/clay microspheres, obtained by OM, prepared with an injection flow rate of 0.150 mL·min^−1^ and airflows of 2.5 and 10 L·min^−1^ are present in Figure 2. These images were analyzed using the software Pixcavator 6.0 and the diameter and volume values were determined. The results obtained are in Table 1.

It is evidenced that the airflow rate of the drag system (2.5 and 10 L·min^−1^) affected the dimensions of the prepared microspheres. The diameter and volume of all microspheres (CS, CS/10%CL and CS/10%AG) decreased when they were manufactured with the higher airflow of the drag system (Table 1). According to Barbosa, et al. [38], the air rate flowing parallel to the needle is the main responsible for the drop drag, preventing their growth. Thus, the higher the airflow rate the larger the drag force, preventing the growth of droplet, resulting in a microsphere with smaller diameter.

The incorporation of the CL and AG clays within chitosan also affected the size of the microspheres. This may be explained on the basis that an increase in viscosity of the chitosan solution with the addition of clays prevents the growth of droplet, resulting in a microsphere with smaller size. The lower dimensions were exhibited by microspheres formed from CS/10%CL as compared to those from CS/10%AG, in the same injection condition. The reason for this is believed to be a consequence of the higher viscosity of the chitosan solution prepared with CL clay due the higher electrostatic interaction between CS and CL, corroborate with WAXD data. The viscosity of the chitosan solution and chitosan/clay suspensions was determined at pH 4.9 with a Brookfield-02 rheometer (Braseq Brasileira de Equipamentos LtdaAv, Jarinu, Brazil) operating at 50 rpm and 20 °C for 10 min. The viscosity measurements were carried out every 30 s; thus, 20 viscosity readings were done for each sample. The viscosity data were evaluated using analysis of variance (ANOVA) and the significance of the model was verified with the F test. In the significant models, the averages were compared with Tukey’s test, with a significance level of 95% (*p* < 0.05) using Sisvar 5.6. According to statistical data, the viscosity of CS solution, CS/10%AG suspension and CS/10%CL suspension were, respectively, 264, 316 and 344 cP. Thus, it is possible that the entrapment efficiency of drugs in CS/10%CL microspheres is greater since an increase in viscosity of the chitosan solution may prevents drug crystals from leaving the droplet. In addition, the increase in viscosity of the chitosan solution, which influences the diffusion of the drug as well as erosion of the microspheres, may result in slowest sustained release [31]. Thus, this sample (CS/10%CL-10), being small in size, have large surface to volume ratios and can be used as a potential carrier for prolonged delivery of drugs, macromolecules and targeted drug delivery.

**Figure 3:**
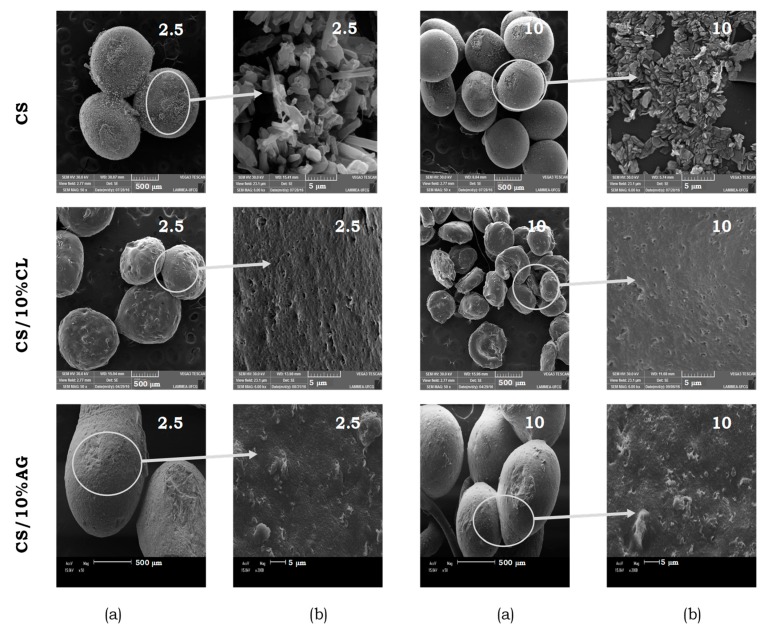
Scanning electron microscopy (SEM) images of chitosan and chitosan/clay microspheres prepared with 10% clay and 2.5 and 10 L·min^−1^ airflow at magnifications (**a**) 50× and (**b**) 2000×.

Figure 3 shows the surface morphology of the chitosan and chitosan/clay microspheres, prepared with 2.5 and 10 L·min^−1^ airflows, at the 50× and 2000× magnifications. All microspheres presented a drop shape with small deformations at the break point; nevertheless, the chitosan/clay microspheres are more elongated, especially that prepared with AG clay (CS/10%AG). In addition, under higher magnification, differences are observed in the morphologies of the microspheres. Chitosan microspheres showed distributed nodular domains (in the form of crystals) along the microspheres, probably due to the presence of agglomerates of polymer chains resulting from the interaction in aqueous medium as reported by Orrego and Valencia [44] in a similar study. Chitosan/clay microspheres presents surfaces free of nodular domains, with a rather rough smooth surface. The dimensions of microspheres decreased when they were manufactured with the higher airflow of the drag system corroborated with OM data. The incorporation of the CL and AG clays within chitosan also affected the size of the microspheres. Compared to chitosan microspheres, the size of chitosan/CL microspheres decreased but the one of chitosan/AG slightly increased.

For all microspheres, voids or porous are observed and by using the Gwyddion 2.50 software, it was possible to calculate the average pore size of the microspheres; the data are in Table 2. The airflow rate of the drag system (2.5 and 10 L·min^−1^) affected the average pore size of the chitosan and chitosan/CL microspheres. The higher airflow rate resulted in the higher mean pore size. On the other hand, this property not change for chitosan/AG microspheres. The incorporation of the CL and AG clays within chitosan also affected the average pore size of the microspheres. In the same injection condition, chitosan/clay microspheres exhibited the smallest pore size, especially the ones prepared with AG clay.

**Figure 4:**
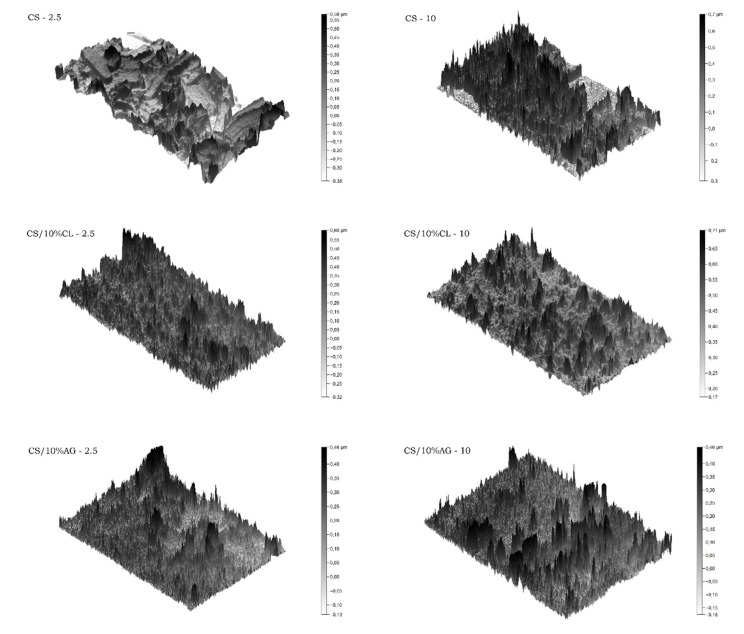
Images of the topography of microspheres, prepared with 2.5 and 10 L·min^−1^ airflow, obtained by Gwyddion 2.50 software.

The topography images, with which it was possible to calculate the data shown in Table 2, using the Gwyddion 2.50 software, of the chitosan and chitosan/clay microspheres (Figure 4) present several peaks that indicate rough and porous morphology of the samples. The darkest and largest peaks refer to the highest roughness. According to these images it is evidenced that the microspheres prepared with CL clay using the higher airflow rate (CS/10%CL-10) presented a more homogeneous morphology relative to pore distribution and surface roughness. Thus, this composition may be more effective for use as controlled drug delivery system. As described by Van de Belt, et al. [45], the total amounts of drug released increase linearly with bulk porosity, pointing to a diffusion mechanism, indicating that the kinetics of drug release are initially controlled to some extent by surface phenomena, while the sustained release over a time span of several days depends on the penetration depth as determined by the bulk porosity of the material. Therefore, by adjusting surface roughness and bulk porosity of microspheres, one can control both the initial as well as the sustained release of the drugs.

**Figure 5:**
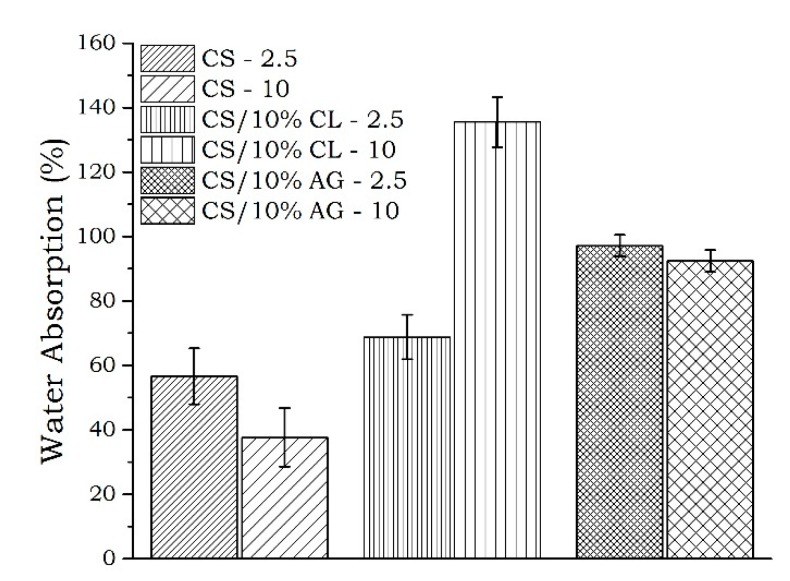
Water absorption of the chitosan and chitosan/clay microspheres at 2.5 and 10 L·min^−1^ airflow.

The water absorption data of the chitosan and chitosan/clay microspheres prepared at the airflow velocities of the 2.5 and 10 L·min^−1^ drag system is shown in Figure 5. The analyses were performed in triplicate and the results of this physical property was calculated as the average of three independent experiments and subjected to statistical analysis. The results were evaluated using analysis of variance (ANOVA) with Tukey’s test (*p* < 0.05) using Sisvar 5.6 and the statistical data are in Table 3.

It was found that the airflow velocities and clay type influenced on water absorption of the microspheres prepared (Figure 5, Table 3). According to statistical data (Table 3), the chitosan microspheres manufactured with CL clay at higher airflow velocities of drag system (CS/10%CL-10) exhibited the higher water absorption (*p* < 0.05) (Table 3). In the same injection condition (10 L·min^−1^), the water absorption of the microspheres decreased in the order CS/10%CL-10 > CS/10%AG-10 > CS-10 (Table 3). This can be due the hydrophilic nature of the clays. Our results are in agreement with result of Lavorgna, et al. [46] that showed chitosan/clay nanocomposite had more water sorption compared to neat chitosan film. Thus, it can be concluded that CS/10%CL-10 microspheres are more indicated to sustained release seeing that this kind of release requires the penetration of dissolution fluids into the interconnecting pores and cracks, which is dictated by the wettability of the polymer matrices and by the number and sizes of the pores in the polymer matrix [45].

**Figure 6:**
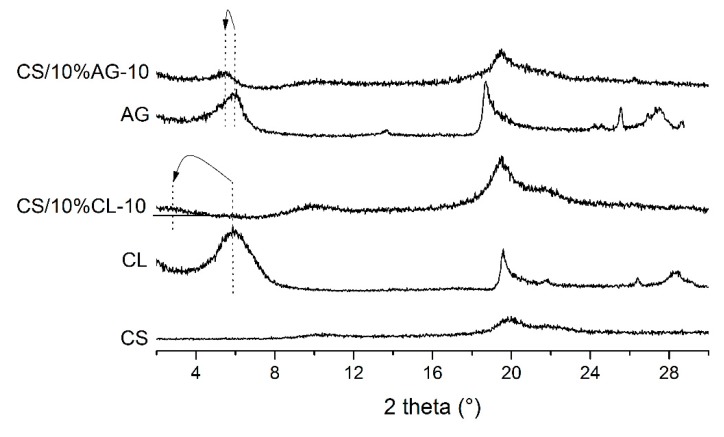
X-ray diffraction (XRD) patterns of chitosan, clays and hybrids chitosan/clay.

Figure 6 shows the wide-angle XRD patterns (WAXD) of chitosan (CS), montmorillonite clay (CL), bentonite clay (AG) and chitosan/clay hybrids (CS/10%CL-10 and CS/10%AG-10). The X-ray diffraction pattern of chitosan (CS) showed a broad band of low intensity between 8–12°, typical of semi-crystalline material, corroborating with the data obtained by Baskar and Kumar [47] and Luo, et al. [48]. As described by Wan, et al. [49], this peak (2*θ* = 10°) corresponds to the crystalline form I of the chitosan structure, this form is orthorhombic and has a unit cell with a = 7.76 Å, b = 10.91 Å and c = 10.30 Å, which is related to the diffraction plane (100). Chitosan also shows a peak in the 20°. These peaks (10° and 20°) are typical fingerprints of chitosan related to hydrated and anhydrous crystals, respectively [50].

Montmorillonite and bentonite clays (CL and AG), presented reflection peaks (001) with approximately 2*θ* at 5.90° and 6.02°, corresponding to a basal interplanar distance (d_001_) of 1.49 nm and 1.47 nm, respectively, characteristic of montmorillonite sodium [51]. After the incorporation of CL and AG within CS, the (001) peak of CL and AG moves to lower angles from 2*θ* = 5.90° to 2*θ* = 2.81° and from 2*θ* = 6.02° to 2*θ* = 5.47°, corresponding to a d_001_ value of 3.14 nm and 1.61 nm for CS/10%CL-10 and CS/10%AG-10, respectively. The increase in the interlayer distance for these nanocomposites was calculated from its corresponding d_001_. Considering the thickness of the montmorillonite layer of 0.96 nm [42], an increase of 2.18 and 0.65 nm in the interlayer spacing for the CS/10%CL-10 and CS/10%AG-10, respectively, was found, indicating the intercalation of bilayers of CS into the interlayers of CL and AG clays and the formation of an intercalated nanostructure. The higher value presented by CS/10%CL-10, suggests that the interlayers of almost all of CL clay were intercalated with bilayers of CS as described by Tan, et al. [52]. The intercalation of bilayer chitosan into the montmorillonite was also obtained by Darder, et al. [53], for chitosan to clay ratios of 5:1 and 10:1. This interaction was possibly favored by the electrostatic interaction of the second layer ($-NH_{3}^{+})$ with the acetate ions of the chitosan solution, which allowed the access to the sites for anion exchange [26].

Due to the fact that clays have different compositions, the clay type also influenced the morphology of the hybrids. The hybrids containing CL clay (CS/10%CL-10) presented the greatest interlayer spacing and a more disordered morphology when compared to hybrids containing AG clay. As a result, it can be assumed that CL clay interacted more strongly with chitosan than AG clay. Since it is generally accepted that electrostatic attractive forces could improve the controlled-drug release properties [54,55,56] and a longer path is required for drug molecules to diffuse out of the more disordered morphology microspheres, it is possible that the CS/10%CL-10 microspheres, may exhibit the subsequent slowest sustained release [57,58].

## 4. Conclusions

Microspheres of chitosan and chitosan/clay were obtained by the precipitation method. The process parameters and the type of clay affected their dimensions (diameter, volume and surface area). Regarding the water absorption and porosity, the clay type had great influence, being montmorillonite (CL clay) responsible for the highest values. In general, the microspheres presented a porous microstructure, being the pore size, percentage of porosity and degree of swelling affected not only by the process parameters but also by the clay type. Chitosan/clay hybrids with intercalated morphology were obtained and the CS/10%CL-10 hybrid presented the greatest interlayer spacing and a more disordered morphology. Thus, it is certain that the chitosan/clay hybrid microspheres prepared with montmorillonite (CL clay) at the airflow velocities of 10 L·min^−1^ drag system can have good drug-controlled release properties. Furthermore, CL clay can provide mucoadhesive and toxin adsorption capabilities, which further suggest strongly that chitosan/CL clay nanocomposite microspheres are quite suitable to be applied in drug-delivery systems in comparison with pure chitosan microspheres. In summary, chitosan–clay nanocomposite is a versatile polymer nanocomposite for biomedical applications, including tissue engineering and controlled drug delivery.

## Figures and Tables

**Table 1:** Microspheres dimensions of chitosan and chitosan/clay.

Sample	Diameter (mm)	Volume (mm^3^)
CS-2.5	1.23 ± 0.10	0.99 ± 0.26
CS-10	0.80 ± 0.05	0.26 ± 0.05
CS/10%CL-2.5	0.56 ± 0.06	0.09 ± 0.03
CS/10%CL-10	0.39 ± 0.06	0.03 ± 0.02
CS/10%AG-2.5	0.88 ± 0.11	0.38 ± 0.13
CS/10%AG-10	0.74 ± 0.10	0.22 ± 0.08

**Table 2:** Average pore size of the chitosan and chitosan/clay microspheres.

Sample	Average Pore Size (nm)
CS-2.5	207
CS-10	358
CS/10%CL-2.5	83
CS/10%CL-10	117
CS/10%AG-2.5	67
CS/10%AG-10	61

**Table 3:** Statistical data for water absorption of the microspheres.

Sample	Water Absorption (%)
CS/10%CL-10	135 A
CS/10%AG-2.5	98 B
CS/10%AG-10	92 B
CS/10%CL-2.5	69 C
CS-2.5	57 CD
CS-10	38 D

## References

[1] Hejazi R., Amiji M. (2003). Chitosan-based gastrointestinal delivery systems. J. Control. Release.

[2] Xue M., Hu S., Lu Y., Zhang Y., Jiang X., An S., Guo Y., Zhou X., Hou H., Jiang C. (2015). Development of chitosan nanoparticles as drug delivery system for a prototype capsid inhibitor. Int. J. Pharm..

[3] Rassu G., Soddu E., Cossu M., Gavini E., Giunchedi P., Dalpiaz A. (2016). Particulate formulations based on chitosan for nose-to-brain delivery of drugs. A review. J. Drug Deliv. Sci. Technol..

[4] Van Woensel M., Wauthoz N., Rosière R., Mathieu V., Kiss R., Lefranc F., Steelant B., Dilissen E., Van Gool S.W., Mathivet T. (2016). Development of siRNA-loaded chitosan nanoparticles targeting Galectin-1 for the treatment of glioblastoma multiforme via intranasal administration. J. Control. Release.

[5] Ilium L. (1998). Chitosan and its use as a pharmaceutical excipient. Pharm. Res..

[6] Remunan-Lopez C., Bodmeier R. (1997). Mechanical, water uptake and permeability properties of crosslinked chitosan glutamate and alginate films. J. Control. Release.

[7] Siafaka P.I., Titopoulou A., Koukaras E.N., Kostoglou M., Koutris E., Karavas E., Bikiaris D.N. (2015). Chitosan derivatives as effective nanocarriers for ocular release of timolol drug. Int. J. Pharm..

[8] Miladi K., Sfar S., Fessi H., Elaissari A. (2015). Enhancement of alendronate encapsulation in chitosan nanoparticles. J. Drug Deliv. Sci. Technol..

[9] Erel G., Kotmakçı M., Akbaba H., Karadağlı S.S., Kantarcı A.G. (2016). Nanoencapsulated chitosan nanoparticles in emulsion-based oral delivery system: In vitro and in vivo evaluation of insulin loaded formulation. J. Drug Deliv. Sci. Technol..

[10] Coppi G., Iannuccelli V. (2009). Alginate/chitosan microparticles for tamoxifen delivery to the lymphatic system. Int. J. Pharm..

[11] Abruzzo A., Cerchiara T., Bigucci F., Gallucci M.C., Luppi B. (2015). Mucoadhesive buccal tablets based on chitosan/gelatin microparticles for delivery of propranolol hydrochloride. J. Pharm. Sci..

[12] Cerchiara T., Abruzzo A., Parolin C., Vitali B., Bigucci F., Gallucci M., Nicoletta F., Luppi B. (2016). Microparticles based on chitosan/carboxymethylcellulose polyelectrolyte complexes for colon delivery of vancomycin. Carbohyd. Polym..

[13] Onishi H. (2010). Chitosan microparticles. J. Drug Deliv. Sci. Technol..

[14] Shu X., Zhu K. (2002). Controlled drug release properties of ionically cross-linked chitosan beads: The influence of anion structure. Int. J. Pharm..

[15] Khlibsuwan R., Siepmann F., Siepmann J., Pongjanyakul T. (2017). Chitosan-clay nanocomposite microparticles for controlled drug delivery: Effects of the MAS content and TPP crosslinking. J. Drug Deliv. Sci. Technol..

[16] Sorby D.L., Liu G. (1966). Effects of adsorbents on drug absorption II: Effect of an antidiarrhea mixture on promazine absorption. J. Pharm. Sci..

[17] Aguzzi C., Cerezo P., Viseras C., Caramella C. (2007). Use of clays as drug delivery systems: Possibilities and limitations. Appl. Clay Sci..

[18] Peppas N. (2004). Devices based on intelligent biopolymers for oral protein delivery. Int. J. Pharm..

[19] Nair L.S., Laurencin C.T. (2007). Biodegradable polymers as biomaterials. Prog. Polym. Sci..

[20] Coviello T., Matricardi P., Marianecci C., Alhaique F. (2007). Polysaccharide hydrogels for modified release formulations. J. Control. Release.

[21] Pongjanyakul T., Khunawattanakul W., Puttipipatkhachorn S. (2009). Physicochemical characterizations and release studies of nicotine–magnesium aluminum silicate complexes. Appl. Clay Sci..

[22] Rojtanatanya S., Pongjanyakul T. (2010). Propranolol–magnesium aluminum silicate complex dispersions and particles: Characterization and factors influencing drug release. Int. J. Pharm..

[23] Khunawattanakul W., Puttipipatkhachorn S., Rades T., Pongjanyakul T. (2008). Chitosan–magnesium aluminum silicate composite dispersions: Characterization of rheology, flocculate size and zeta potential. Int. J. Pharm..

[24] Khunawattanakul W., Puttipipatkhachorn S., Rades T., Pongjanyakul T. (2010). Chitosan–magnesium aluminum silicate nanocomposite films: Physicochemical characterization and drug permeability. Int. J. Pharm..

[25] Khunawattanakul W., Puttipipatkhachorn S., Rades T., Pongjanyakul T. (2011). Novel chitosan–magnesium aluminum silicate nanocomposite film coatings for modified-release tablets. Int. J. Pharm..

[26] Yuan Q., Shah J., Hein S., Misra R. (2010). Controlled and extended drug release behavior of chitosan-based nanoparticle carrier. Acta Biomater..

[27] Ali A., Ahmed S. (2018). A review on chitosan and its nanocomposites in drug delivery. Int. J. Biol. Macromol..

[28] Pereda M., Amica G., Rácz I., Marcovich N.E. (2011). Structure and properties of nanocomposite films based on sodium caseinate and nanocellulose fibers. J. Food Eng..

[29] Bernkop-Schnürch A., Dünnhaupt S. (2012). Chitosan-based drug delivery systems. Eur. J. Pharm. Biopharm..

[30] Hu L., Sun Y., Wu Y. (2013). Advances in chitosan-based drug delivery vehicles. Nanoscale.

[31] Sinha V., Singla A.K., Wadhawan S., Kaushik R., Kumria R., Bansal K., Dhawan S. (2004). Chitosan microspheres as a potential carrier for drugs. Int. J. Pharm..

[32] Kuo S.M., Niu G.C.C., Chang S.J., Kuo C.H., Bair M.S. (2004). A one-step method for fabricating chitosan microspheres. J. Appl. Polym. Sci..

[33] Jameela S., Jayakrishnan A. (1995). Glutaraldehyde cross-linked chitosan microspheres as a long acting biodegradable drug delivery vehicle: Studies on the in vitro release of mitoxantrone and in vivo degradation of microspheres in rat muscle. Biomaterials.

[34] He P., Davis S.S., Illum L. (1999). Chitosan microspheres prepared by spray drying. Int. J. Pharm..

[35] Denkbaş E.B., Kilicay E., Birlikseven C., Öztürk E. (2002). Magnetic chitosan microspheres: Preparation and characterization. React. Funct. Polym..

[36] Wang L.-Y., Gu Y.-H., Zhou Q.-Z., Ma G.-H., Wan Y.-H., Su Z.-G. (2006). Preparation and characterization of uniform-sized chitosan microspheres containing insulin by membrane emulsification and a two-step solidification process. Colloids Surf. B.

[37] Wang L.-Y., Ma G.-H., Su Z.-G. (2005). Preparation of uniform sized chitosan microspheres by membrane emulsification technique and application as a carrier of protein drug. J. Control. Release.

[38] Barbosa H.D.C., Santos B.F.F.D., Tavares A.A., Barbosa R.C., Fook M.V.L., Canedo E.L., Silva S.M.D.L. (2018). Inexpensive Apparatus for Fabricating Microspheres for 5-Fluorouracil Controlled Release Systems. Int. J. Chem. Eng..

[39] Il’Ina A., Varlamov V. (2004). Hydrolysis of chitosan in lactic acid. Appl. Biochem. Microbiol..

[40] Brugnerotto J., Lizardi J., Goycoolea F., Argüelles-Monal W., Desbrieres J., Rinaudo M. (2001). An infrared investigation in relation with chitin and chitosan characterization. Polymer.

[41] Leite I.F., Soares A.P., Carvalho L.H., Raposo C.M.O., Malta O.M.L., Silva S.M.L. (2009). Characterization of pristine and purified organobentonites. J. Therm. Anal. Calorim..

[42] Utracki L.A. (2004). Clay-Containing Polymeric Nanocomposites.

[43] Depan D., Kumar A.P., Singh R.P. (2006). Preparation and characterization of novel hybrid of chitosan-*g*-lactic acid and montmorillonite. J. Biomed. Mater. Res. A.

[44] Orrego C.E., Valencia J.S. (2009). Preparation and characterization of chitosan membranes by using a combined freeze gelation and mild crosslinking method. Bioproc. Biosyst. Eng..

[45] Van de Belt H., Neut D., Uges D., Schenk W., Van Horn J., Van der Mei H., Busscher H. (2000). Surface roughness, porosity and wettability of gentamicin-loaded bone cements and their antibiotic release. Biomaterials.

[46] Lavorgna M., Piscitelli F., Mangiacapra P., Buonocore G.G. (2010). Study of the combined effect of both clay and glycerol plasticizer on the properties of chitosan films. Carbohyd. Polym..

[47] Baskar D., Kumar T.S. (2009). Effect of deacetylation time on the preparation, properties and swelling behavior of chitosan films. Carbohyd. Polymers.

[48] Luo D., Sang L., Wang X., Xu S., Li X. (2011). Low temperature, pH-triggered synthesis of collagen–chitosan–hydroxyapatite nanocomposites as potential bone grafting substitutes. Mater. Lett..

[49] Wan Y., Creber K.A., Peppley B., Bui V.T. (2003). Ionic conductivity of chitosan membranes. Polymer.

[50] Baklagina Y., Klechkovskaya V., Kononova S., Petrova V., Poshina D., Orekhov A., Skorik Y. (2018). Polymorphic Modifications of Chitosan. Crystallogr. Rep..

[51] Paiva L.D., Morales A., DÍAZ F.V. (2008). Argilas organofílicas: Características, metodologias de preparação, compostos de intercalação e técnicas de caracterização. Cerâmica.

[52] Tan W., Zhang Y., Szeto Y.-S., Liao L. (2008). A novel method to prepare chitosan/montmorillonite nanocomposites in the presence of hydroxy-aluminum oligomeric cations. Compos. Sci. Technol..

[53] Darder M., Colilla M., Ruiz-Hitzky E. (2003). Biopolymer–clay nanocomposites based on chitosan intercalated in montmorillonite. Chem. Mater..

[54] Liu W.G., Li F., Zhao X.D., Yao K.D., Liu Q.G. (2002). Atom force microscopic characterisation of the interaction forces between bovine serum albumin and cross-linked alkylated chitosan membranes in media of different pH. Polym. Int..

[55] Perugini P., Genta I., Conti B., Modena T., Pavanetto F. (2003). Periodontal delivery of ipriflavone: New chitosan/PLGA film delivery system for a lipophilic drug. Int. J. Pharm..

[56] Liu K.-H., Liu T.-Y., Chen S.-Y., Liu D.-M. (2008). Drug release behavior of chitosan–montmorillonite nanocomposite hydrogels following electrostimulation. Acta Biomater..

[57] Wang X., Du Y., Luo J., Lin B., Kennedy J.F. (2007). Chitosan/organic rectorite nanocomposite films: Structure, characteristic and drug delivery behaviour. Carbohyd. Polym..

[58] Lin N., Huang J., Chang P.R., Feng L., Yu J. (2011). Effect of polysaccharide nanocrystals on structure, properties and drug release kinetics of alginate-based microspheres. Colloids Surf. B.

